# Intranasal sedation using ketamine and midazolam for pediatric dental treatment (NASO): study protocol for a randomized controlled trial

**DOI:** 10.1186/s13063-017-1919-2

**Published:** 2017-04-11

**Authors:** Heloisa Sousa Gomes, Analya Rodrigues Miranda, Karolline Alves Viana, Aline Carvalho Batista, Paulo Sucasas Costa, Anelise Daher, Geovanna de Castro Morais Machado, Joji Sado-Filho, Liliani Aires Candido Vieira, Patrícia Corrêa-Faria, Marie Therese Hosey, Luciane Rezende Costa

**Affiliations:** 1 Dentistry Graduate Program, Faculdade de Odontologia (FO), Universidade Federal de Goiás (UFG), Goiânia, GO 74605-220 Brazil; 2 Department of Stomatology (Oral Pathology), FO/UFG, Goiânia, GO 74605-220 Brazil; 3 Department of Pediatrics, Faculdade de Medicina (FM), UFG, Goiânia, GO 74605-020 Brazil; 4 Health Sciences Graduate Program, FM/UFG, Goiânia, GO 74605-020 Brazil; 5 Department of Oral Health, FO/UFG, Goiânia, GO 74605-220 Brazil; 6 Pediatric Dentistry, Division of Population and Patient Health, King’s College London Dental Institute, Bessemer Road, London, SE5 9RS UK; 7 Faculdade de Odontologia, Universidade Federal de Goiás, Primeira Avenida, Setor Universitário, CEP: 74605-220 Goiânia, Goiás Brazil

**Keywords:** Dental care for children, Conscious sedation, Child behavior, Midazolam, Ketamine, Administration intranasal, Pain assessment, Amnesia, Stress, Physiological, Patient satisfaction

## Abstract

**Background:**

Uncooperative children may need to receive dental treatment under sedation, which is indicated when nonpharmacological behavior guidance is unsuccessful. There are randomized controlled trials (RCTs) comparing different sedative protocols for dental procedures; however, the evidence for superiority of one form over another is weak. The primary aim of this study is to investigate the efficacy of intranasally administered ketamine plus midazolam for the dental treatment of children.

**Methods:**

We have designed a three-armed, parallel RCT to assess intranasal sedation using ketamine/midazolam in terms of the following measures: efficacy, safety, and cost-effectiveness. Two- to 6-year-old healthy children, referred for dental treatment in a dental sedation center in Brazil due to uncooperative behavior and requiring restorative dental procedures, will be recruited. Each child will be randomly assigned to one of the three groups: A – Intranasal administration of ketamine (4.0 mg/kg, maximum 100 mg) and midazolam (0.2 mg/kg, maximum 5.0 mg); B – Oral administration of ketamine (4.0 mg/kg, maximum 100 mg) and midazolam (0.5 mg/kg, maximum 20 mg); and C – Oral administration of midazolam (1.0 mg/kg, maximum 20 mg). The primary outcome is the child’s behavior assessed through an observational scale using digital videos of the restorative dental treatment under sedation. The secondary outcomes are as follows: acceptance of sedative administration; memory of intraoperative events; the child’s stress; adverse events; the child’s pain during the procedure; the parent’s, dentists’, and child’s perceptions of sedation; and economic analysis. Measures will be taken at baseline and drug administration and during and after the dental procedure. The necessary sample size was estimated to be 84 children after a blinded interim analysis of the first 30 cases.

**Discussion:**

This study will provide data that can substantially add to science and pediatric dentistry as it examines the effect of sedative regimes from different perspectives (outcomes).

**Trial registration:**

ClinicalTrials.gov, identifier: NCT02447289. Registered on 11 May 2015, named “Midazolam and Ketamine Effect Administered Through the Nose for Sedation of Children for Dental Treatment (NASO).”

**Electronic supplementary material:**

The online version of this article (doi:10.1186/s13063-017-1919-2) contains supplementary material, which is available to authorized users.

## Background

Preschool children may present dental behavior management problems (DBMP) and, therefore, refuse routine dental treatment due to several factors such as dental fear [1] and personal temperament [2]. Consequently, that group of patients are more likely to be referred for a dental sedation appointment [3–5] or general anesthesia [6].

Conscious sedation is a form of advanced behavior guidance technique, which is indicated for uncooperative or fearful/anxious children due to a lack of psychological, emotional or mental maturity or a physical or medical disability [7]. This pharmacological approach aims to enhance the patient’s physical comfort and safety and to control anxiety and behavior, as well as to allow for the completion of the procedure [7]. Several randomized controlled trials (RCTs) have been published demonstrating the efficacy of conscious sedation in pediatric dentistry, but evidence on this topic, though still weak, points to the efficacy of midazolam [8].

Our research group has conducted investigations aiming to find the most beneficial sedative protocol for young children undergoing dental treatment. In one of these studies, children under 36 months displayed more cooperative behavior after oral administration of midazolam (0.5 mg/kg) plus ketamine (3.0 mg/kg) compared to either oral midazolam (1.0 mg/kg) or no sedative agent (placebo) [9]. Therefore, we concluded that, for young children, it might be advisable to combine ketamine with midazolam to provide better results in pediatric dental sedation.

The research concerning intranasal procedural sedation has been highlighted due to its faster onset of action and recovery time and less discomfort and cost compared to other routes of sedative administration [10]. In line with the aforementioned efficacy of orally administered ketamine/midazolam [9], we did a search in PubMed and found only one study in pediatric dentistry that used intranasally administered ketamine plus midazolam [11]. Based on a crossover design with 45 children aged 2–6 years, that study [11] revealed high success rates for intranasal sedation as follows: ketamine (6.0 mg/kg) – 89%; midazolam and ketamine (0.2 mg/kg and 4.0 mg/kg) – 84%; and midazolam (0.3 mg/kg) – 69%. In the medical pediatric field, the combination of ketamine and midazolam to perform gastric aspirates has been successful [12]. However, there is a lack of RCTs investigating the intranasal route to deliver ketamine-midazolam in procedural sedation.

Pediatric dental sedation outcomes have primarily been assessed through children’s behavior during the procedure, but the assessment of other “core variables,” including baseline anxiety, completion of treatment, and patient satisfaction or preference, is advisable [8]. However, there are other assessments that can be beneficial for the evaluation of sedation success if performed using a systematic method. Given the lack of evidence on which a sedative regimen is more effective for pediatric dental patients and the requirement for more well-designed studies [8], the development of a RCT on pediatric dental sedation comprising multiple assessments is timely. For the purpose of this study, multiple assessments are defined as specified in Table 1.

**Table 1:** Definitions of the multiple outcomes included in this study protocol

Outcome	Definition	Instrument/tool
Children’s behavior toward dental treatment	Crying and movement during the dental sedation	Ohio State University Behavioral Rating Scale (OSUBRS) [13]
Children’s pain during the dental sedation	Facial expression of pain during the dental procedure under sedation	The Faces, Legs, Activity, Cry, Consolability Pain Assessment Tool (FLACC) [14]
Children’s acceptance of the sedative administration	Crying, movement and pain during the sedative administration through the oral and intranasal routes	OSUBRS [13] and FLACC [14]
Memory of intraoperative procedures	Recall/recognition of pictures and events that occurred throughout dental appointment	Three-stage procedure [15] on the treatment day and semistructured interview with parents on the next day
Children’s, parents’, and dentists’ stress	Physiological response to stress during dental treatment under conscious sedation	Cortisol levels in saliva of all participants and self-report from parents and dentists
Children’s perceptions of sedation	How do the children perceive the dental sedation procedure?	Semistructured qualitative interview 1 week later
Parents’ and dentists’ perceptions of children’s sedation	If parents and dentists are satisfied, feel stress or think that children had pain during the dental sedation	Questions answered through a Visual Analog Scale (VAS)
Adverse events	Unexpected and undesirable response to sedatives that threaten or cause patient injury or discomfort [16]	World SIVA International Sedation Task Force Tool [16]
Economic analysis	Cost-effectiveness	Decision tree [17]

Thus, the overall aim of this paper is to present the methodology of a RCT to investigate the efficacy of intranasally administered ketamine plus midazolam compared to the same drugs administered orally and to a control sedative (orally administered midazolam only). The specific aims are to investigate the following outcomes: the children’s behavior during the dental sedation procedure (primary outcome); the acceptance of the sedative administration; the pain and memory of intraoperative events; the children’s, parents’, and dentists’ perceptions of sedation and stress; the occurrence of adverse events during and after sedative administration; and the economic analysis of the three sedative regimes.

The primary hypothesis is that the combination of ketamine and midazolam, administered intranasally, is more efficacious when compared to the same combination or midazolam only administered orally as measured by the child’s behavior according to the Ohio State University Behavioral Rating Scale (OSUBRS). Sedation is considered efficacious if quiet behavior (OSUBRS score 1) occurs at or above 60% of the session length.

## Methods/design

### Ethical considerations

Ethical approval was granted by the Research Ethics Committee of the Universidade Federal de Goiás (UFG), Brazil (protocol #857.066, 12 November 2014). This study is registered in the Clinical Trials database (http://clinicaltrials.gov/ct2/show/NCT02447289, NCT02447289 (see Additional file 1). If changes are required at any stage of the study, the alterations will be recorded in the protocol in the Clinical Trials database and reported to the Ethics Committee. Informed consent will be sought from the parents of the children. The process of obtaining informed consent will consist of the reading aloud of consent by a research participant sitting next to at least one of the child’s parents; after reading the Consent Form (Additional file 2), the researcher will encourage the parent to ask any remaining questions; when the parent feels able to make a decision (in the same or in another session), they will be asked about the decision to participate. As there are data that will be collected from parents and dentists, they will also be invited to participate and, if in agreement, they will sign a specific Consent Form (Additional file 2). Participants can withdraw from the study at any time, and withdrawal from the study will not affect their dental care in the dental school. The children who participate in the study who present more dental treatment needs, or those whose parents refuse to participate in the study, will also be treated in the dental school. All collected data (questionnaire answers, scales, digital videos, saliva, etc.) involving children, parents, and dentists will be kept confidential by the team, which will adopt password-encrypted access to databases and the use of code instead of the participant’s name. The results, whether favorable or not, will be made public through the presentation of abstracts at events and the publication of articles.

### Study design and setting

This study is a three-armed, triple-blind RCT with a parallel design performed at the UFG Dental School in the Dental Sedation Center Núcleo de Estudos em Sedação Odontológica (NESO) (translation: Study Center in Dental Sedation). NESO is the only public outpatient dental sedation center available in central Brazil and is comprised of a multidisciplinary team that provides dental sedation for referred people, mostly children. NESO follows sedative routines recommended by international guidelines [18]. Each member of the research team is trained to perform a different childcare role under sedation, and the team consists of pediatric dentists, a pediatrician, an anesthesiologist, a psychologist, general dentists, and graduate and undergraduate students.

**Fig. 1:**
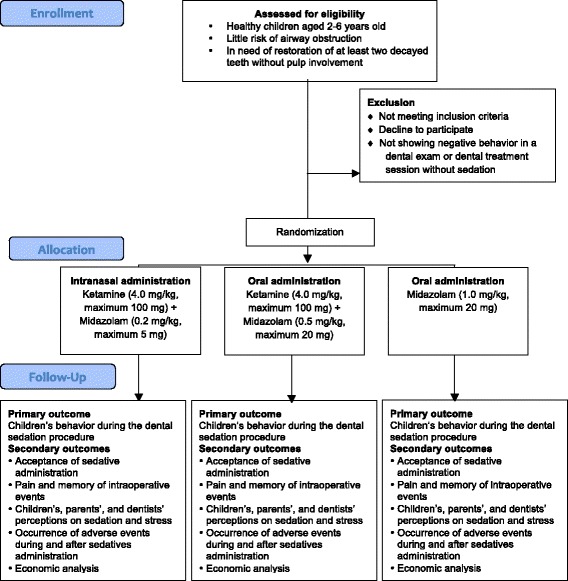
Flow diagram of the intranasal sedation using ketamine and midazolam for pediatric dental treatment (NASO) study protocol

**Fig. 2:**
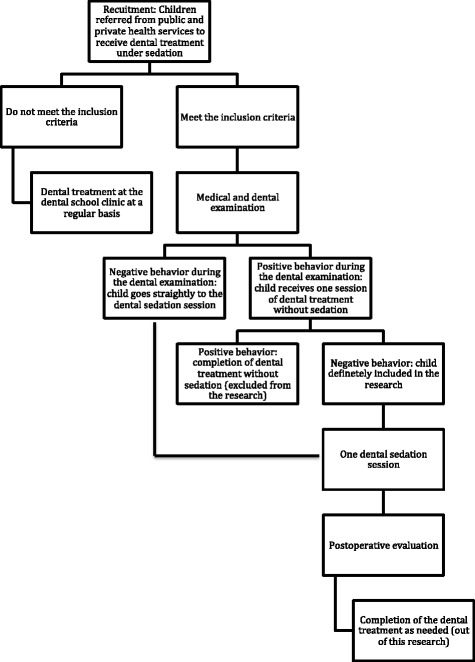
A closer view of the children’s participation flow in the intranasal sedation using ketamine and midazolam for pediatric dental treatment (NASO) study protocol

**Fig. 3:**
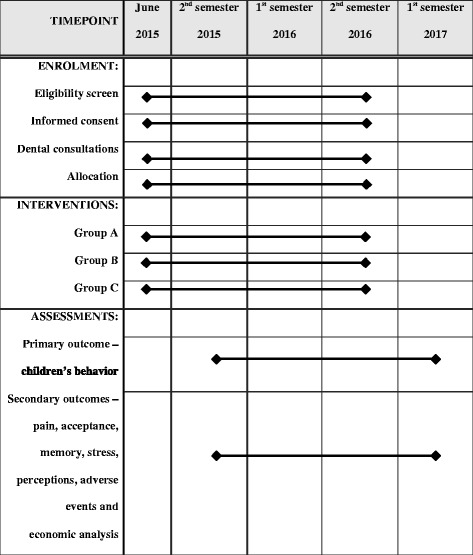
Schedule of the enrollment, interventions, and assessments

The development of this study is shown in a flow diagram (Figs. 1 and 2) and will be carried out according to a timeline (Fig. 3). This flow is detailed in the subsequent subheadings and is in agreement with the “Standard Protocol Items: Recommendations for Interventional Trials” (SPIRIT) Statement (Additional file 3) [19]. Members of the research team were trained for each step of the study.

A pilot study was performed in December 2014 to rehearse the recruitment, randomization, allocation, documentation, and sedation procedures according to the already planned methods. In this phase, nine patients were recruited, and three received dental treatment with sedation; however, they will not be included in the group of participants of the final sample.

### Participants and recruitment

Healthy preschool children, referred for dental treatment under sedation at NESO due to uncooperative behavior requiring dental restorative procedures, will be assessed for the eligibility criteria in a recruitment appointment.

The inclusion criteria for the children are as follows: aged 2–6 years old with a physical status categorized as ASA I (healthy) or II (mild systemic disease) [20] and little risk of airway obstruction [20] (Mallampati I and II and tonsil hypertrophy occupying less than 50% of the oropharynx) [21], no medical history of neurological or cognitive alterations, absence of facial deformities, were born at term, do not use medications that may impair cognitive functions, and have at least two decayed teeth without pulp involvement, requiring dental restoration under local anesthesia and rubber dam. If there are systemic alterations that contraindicate sedation during the research, treatment will be postponed or interrupted.

The exclusion criterion is related to the child’s favorable behavior in a dental restorative session without sedation conducted by the pediatric dentist assisted by other members of the research team. Therefore, if a child shows positive behavior (acceptance of treatment, is willing to collaborate with the dentist and follow-up on the dentist’s recommendations) [22] or definitely positive behavior (interacts with the dentist, interested in the dental procedure and has a fun time) [22] in that session, they will be excluded from the study. This criterion was proposed to avoid inclusion of cooperative children in the sedation session, thereby introducing bias in the final results.

### Randomization and blinding

One blinded researcher (LRC) will carry out the randomization using the online calculator (http://www.randomization.com) that will determine the intervention group for each participant, using blocks of 15 cases. An allocation concealment strategy is achieved with a specific code enclosed in sequentially numbered, opaque, sealed envelopes to conceal the sequence until the intervention is assigned; numbers N1…N84 are written in the envelopes in advance, and the envelopes are opened sequentially, only after the participant’s name is written on the appropriate envelope.

All participants, as well as the researchers who analyze the measures throughout the data collection, will be blinded to the intervention group. Only the pediatrician (PSC) and the anesthesiologist (JSF) will be aware of the group allocation to be able to take urgent measures in case of adverse events. The statistician will also be blinded during the analysis, and only after data collection is completed and the initial analyses are performed, the randomization code will be broken to input the group allocation.

### Interventions

**Fig. 4:**
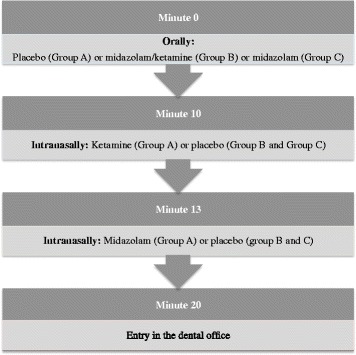
Sequence of sedative administration

Children who are included in the study will be randomly assigned to one of the three comparison groups (Table 2). The administration of sedatives follows a strict sequence and time interval (Fig. 4), and no supplemental nitrous oxide/oxygen will be used.

**Table 2:** Interventions according to groups

Group	Type	Intranasal	Oral
A	Experimental	Ketamine (4.0 mg/kg, maximum 100 mg)^a^ + midazolam (0.2 mg/kg, maximum 5.0 mg)^b^	Placebo
B	Drug route comparison	Placebo	Ketamine (4.0 mg/kg, maximum 100 mg)^a^ + midazolam (0.5 mg/kg, maximum 20 mg)^c^
C	Control	Placebo	Midazolam (1.0 mg/kg, maximum 20 mg)^c^

^a^Injectable solution in a concentration of 50.0 mg/mL (Ketamin S, Cristalia, Sao Paulo, Brazil

^b^Injectable solution in a concentration of 5.0 mg/mL (Dormire solução injetável, Cristalia, Sao Paulo, Brazil)

^c^Oral solution in a concentration of 2.0 mg/mL (Dormire solução oral, Cristalia, Sao Paulo, Brazil)

The sedative administration will be as follows:

1. The anesthesiologist will confirm the child’s health status and fasting time; if there is any problem, the procedure will be re-scheduled and postponed for at least 1 week
2. The child and a parent will be positioned in a dental chair in the sedative delivery room
3. The administration of the sedative agent will be filmed with one action camera mounted on the anesthesiologist’s head while an observer records the heart rate and oxygen saturation during administration
4. The anesthesiologist will first administer the syrups (midazolam/ketamine, midazolam or placebo) per the oral route (Fig. 4); if the child does not spontaneously accept the syrups, a mouth prop and active physical restraint by the parent will be used
5. The anesthesiologist will administer the sedatives or placebo per the intranasal route (Fig. 4), starting with ketamine dispensed in an insulin syringe and an atomizer (LMA MAD Nasal, Teleflex, Fort Worth, TX, USA) with a maximum dose of 0.5 mL per nares and followed by midazolam
6. The child will be allocated to the intervention group (A, B or C) according to the previous randomization (Table 2)

Because the participants and research team, other than the anesthesiologist and pediatrician, are blinded to the intervention, the oral and intranasally administered placebos will be prepared as magistral formulations with the same characteristics as those of the active drug.

### Medical and dental examination

The medical examination will be performed to confirm the health history and obtain vital signs at baseline. For the standard dental examination, a dentist will perform a dental prophylaxis procedure and intraoral examination, while another dentist or dental student records the dental needs in a specific form according to the recommendations from the World Health Organization [23]. During this procedure, nonpharmacological behavior management methods will be used as necessary. If the child is cooperative (positive or definitely positive behavior according to the Frankl scale [22]), they will be scheduled for one dental session treatment without sedation; otherwise, they will go directly to a dental session treatment under sedation.

### Dental session treatment with or without sedation

During the dental examination, the child may present positive or negative behavior. If positive, the child will be scheduled for a restorative dental treatment session without sedation to confirm the need for sedation. If the behavior is not positive, the child will be scheduled directly for pediatric dental sedation. Both restorative treatment sessions will be as similar as possible and are detailed below. A pediatric dentist will perform one tooth restoration under local anesthesia using a rubber dam. Nonpharmacological behavior management methods will be used as necessary.

The procedures for data collection will be carried out according to the following steps:

#### Saliva sample collection

Saliva samples will be collected from the child, pediatric dentist, and parent at the following times:

1. Just upon arrival at the dental school
2. *For the sedation session only, 25 min after the administration of the last sedative from the child only*
3. Twenty-five minutes after local anesthesia
4. Twenty-five minutes after the end of the procedure

The collection time is 25 min after the stressful stimulus because of the time required for the cortisol level to reach its peak in the saliva [24].

The collection of saliva samples will be conducted using Salivette tubes (Sarstedt Inc., Nümbrecht, Alemanha) with gloved hands. A cotton roll will be placed in the child’s mouth for a period of 1 to 2 min, until it is soaked with saliva. Then, the cotton will be returned to the tube and centrifuged. The saliva sample will then be kept frozen until the analysis procedure.

#### Memory assessment

1. Before starting the dental procedure, two pictures will be shown to the child through a tablet with an interval of 4 min. The child must verbally identify the image. If they do not identify the image or cannot name the item displayed, the researcher will speak the name and ask the child to repeat it (phase 1 – encoding phase)
2. *For the sedation session only, the two different figures will be shown 1 and 5 min after the administration of the last sedative (minute 13)*
3. Immediately before the dental prophylaxis procedure, the dentist will show an animal toy for the child and ask them to verbally identify it (“There is a bug here in your tooth. Do you know what it looks like? This here. What is this?”)
4. Immediately before discharge, the dentist will ask the child to speak the names of all of the items in phase 1 (recall task) and identify the pictures presented in the encoding phase among four figures – two target and two distractor pictures (recognition task). Similarly, the child will be asked which animal toy was shown before the dental prophylaxis procedure (phase 2 – recognition and recall tasks)

The times of the presentation of the pictures in this three-stages procedure (encoding, retention interval and test phase) [15] were chosen based on another study in pediatric dentistry [25].

#### Dental restorative procedure

1. One parent will sit in the dental chair with the child’s legs supported on their lap
2. The dental team will consist of the pediatric dentist and a dental assistant; *in the sedation session, there will be a third person who is trained to monitor the child, called the “observer”*
3. Video recording: an action camera will be positioned at the head of the operator to record the child’s face and body up until the anesthetic procedure, and another camera mounted on a tripod will record the whole scenario during the entire dental appointment
4. The pediatric dentist will perform the dental prophylaxis procedure at low speed in a front tooth without caries. This procedure is proposed as a baseline, non-painful dental stimulus, for the comparison of the occurrence and intensity of pain during dental treatment
5. The dentist will execute the anesthetic procedure (topic and infiltrative)
6. The dentist will perform the dental restorative procedure with composite or glass ionomer cement in one molar tooth after preparing the tooth with a high speed handpiece, removing the caries using excavators and under rubber dam isolation
7. At the end of the session, the dentist and the dental assistant will assess the child’s behavior according to the Frankl scale [22]

The pediatric dentists are trained in the application of the Frankl scale [22], which classifies the child’s behavior as follows: (1) Definitely negative – refusal of treatment; intense crying, fear or any other evidence of extreme negativism, (2) Negative – reluctance to accept treatment; lack of cooperation; any other negative attitude, (3) Positive – acceptance of treatment; willingness to cooperate with the dentist despite some caution; followed instructions from the dentist, (4) Definitely positive – good behavior toward the dentist interested in dental procedures; had fun with the situation.

The unsedated child will be scheduled for the next phase of research (treatment under sedation) if they present negative or definitely negative behavior according to the Frankl scale [22]. In this case, the child’s parent will receive preoperative guidelines which include fasting instructions. On the other hand, if the unsedated child displays positive or definitely positive behavior, they will be referred for dental care using nonpharmacological approaches in the dental school.

Dental treatment will be aborted if the child presents definitely negative behavior anytime during a given session. Additionally, if any adverse event happens, the dental treatment session will be interrupted or aborted, depending on the seriousness of the episode [16]. Information about positive or definitely negative behavior and treatment being aborted will be recorded for further analysis.

After the conclusion of the dental restoration in the sedated child, the observer will take the child and their parent to the postanesthetic recovery room, where the parent and dentist will independently answer questions about their perception of the sedation success, the child’s pain during the procedure, and their own stress during the procedure by answering three questions through a 10-cm VAS: (1) How do you assess the child sedation? (2) How much stress did you feel during treatment? (3) How much pain do you think the child felt during treatment?

The child/parent will be discharged after fulfilling the recommended criteria [18] and if additional dental procedures are required, they will be scheduled for treatment at NESO.

### Postoperative assessments

Twenty-four hours after dental restoration under sedation, a member of the research team will call the child’s parent to obtain information on adverse events and memory. Mothers are asked to answer the following questions: (1) Do you think your son/daughter remembers the performed interventions? Why? and (2) Did your son/daughter say something regarding the performed interventions? If yes, what? These answers will help to identify the sedative’s amnesic ability.

Intra and postoperative adverse events will be registered as recommended by the World SIVA International Sedation Task Force as minimal, minor, and sentinel risks [16]. The event report tool also allows for the registration of the interventions performed to treat the adverse events, the outcome of the adverse events and, finally, the assignment of an overall severity for the sedation encounter.

One week after the dental restoration under sedation, children older than 3 years will be asked about their perceptions of the dental sedation by means of a semistructured interview. The researchers will conduct this interview through an illustrated history called “Peppa goes to the dentist.” In each history scenario, the child will be asked questions related to their perceptions of previous dental visits such as, “Did you go to the dentist? How do you feel waiting for the dentist? How do you feel when you sit in a dental chair? Did you drink any syrup in the dentist? How do you feel after drinking this syrup? Did the dentist touch your mouth?” The interviews are video recorded. After the interviews, they are transcribed verbatim and analyzed by content analysis using the software NVivo (QSR International Pty Ltd., Melbourne, VIC, Australia).

### Saliva analysis

After collecting the saliva samples, the tubes will be centrifuged at 3000 rpm for 15 min at the Immunohistochemistry and Analysis of Saliva Laboratory. Until the moment of its analysis, the saliva samples will be stored frozen and coded to protect participant confidentially. Laboratory analysis to measure cortisol in saliva samples will be performed using an Enzyme Immunoassay Kit (Salimetrics Kit, LLC, Carlsbad, CA, USA) for salivary cortisol, following the information that came with the same kit. A photometer reader (Perlong; DNM – 9602 Microplate Reader, Buena Park, CA, USA) at an absorbance of 450 nm will be used for analysis of the cortisol values obtained. Cortisol level is determined in accordance with standard curves prepared according to the manufacturer (Salimetrics Kit, LLC, Carlsbad, CA, USA), with a detection limit of 3000 μg/dL to 0.012 μg/dL.

### Analyses of the video records

At the Qualitative Research Laboratory, observers (dental undergraduate students) blinded to the intervention will be trained and calibrated to assess children’s behavior in the video files, according to the OSUBRS [13] score, using the software The Observer XT (Noldus, The Netherlands), and the FLACC [14].

OSUBRS scores are 1 for quiet behavior, 2 for crying without movement, 3 for movement without crying, and 4 for struggling. Observers will continuously assign OSUBRS scores while watching the videos of the dental treatment during the length of the procedure. The software calculates the percentage for each score in a given session.

Pain during administration of sedation and local anesthesia will be assessed in videos by the FLACC, which has five categories (Face, Legs, Activity, Cry, Consolability) that are scored from 0 to 2 and result in a total score of 10 [14]. Children will be observed for the whole period of each phase of the administration of sedatives as well as during the administration of the dental local anesthetic (local puncture for infiltrative anesthesia, injection of anesthetic, and after removal of the carpule syringe), and a score for each category will be assigned. In addition, for comparison with potentially not painful procedures, videos will be assessed for two other specific periods: the start of the dental treatment and during the prophylaxis procedure in a sound deciduous tooth.

One trained and calibrated observer will analyze each video. The same observer will review one in five videos again after 2 weeks to evaluate the intraobserver agreement.

### Economic analysis

The purpose of this analysis is to conduct a cost-effectiveness analysis comparing the sedation regimes for children during dental procedures, from the perspective of Brazil’s public health system. The primary outcome will be the child’s behavior and adverse events during and after sedative administration. The preoperative procedures are inherent to all regimes, and their costs will not be evaluated. The direct medical costs will be taken into consideration. A decision tree will be developed using MS Excel (Microsoft, Redmond, WA, USA) to estimate the costs and benefits associated with the techniques. An incremental cost-effectiveness ratio (ICER) will be carried out to classify the different strategies [17, 26].

### Outcome measures

The experimental, comparison and control groups will be compared regarding one primary and several secondary outcomes. The main outcome is the child’s behavior assessed according to the OSUBRS. Based on this outcome, the dental sedation session will be considered efficacious if the child’s behavior remains quiet (OSUBRS score 1) for 60% or above of the session length since the planned restorative treatment has been completed. The secondary outcomes are as follows:

1. Sedative administration acceptance: this outcome will be assessed according to the child’s behavior and pain. An OSUBRS score of 1 (quiet) for over 60% of the administration duration represents a good acceptance (dichotomous variable, yes or no). The presence or absence of pain and the pain intensity are determined with the FLACC
2. Memory of intraoperative events: amnesia is desirable and means that children do not recall the figures/events after sedative administration (dichotomous variable, yes or no), on the same day, 24 h or 1 week later
3. Salivary cortisol levels: children’s, parents’ and dentists’ stress will be evaluated by salivary cortisol levels, which are determined by the enzyme-linked immunosorbent assay test as described elsewhere [27] (continuous variable)
4. Adverse events: intra and postoperative adverse events will be assessed through the World SIVA Tool and categorized as minor, sentinel, and major [16] but analyzed as yes/no for the purpose of this study
5. Pain during treatment: the presence or absence of pain and its intensity will be assessed through the FLACC [14]
6. Caregivers’ and dentists’ perceptions of sedation: the perceptions in the sedative groups will be compared according to the answers in the Visual Analog Scale (VAS) (continuous variable)
7. Child’s perception of dental sedation: this will be assessed through content analysis of the transcripts of a semistructured qualitative interview
8. Cost analysis: the cost of each sedation protocol according to the methodology used will be analyzed and compared across groups

#### Strategies to improve adherence to research

In order to maximize follow-up and adherence to the study, the following strategies were adopted:

- During recruitment, the participants most likely to join the study were selected. This was verified from the attendance of the participants in the consultations of clinical examination and dental treatment without sedation. Individuals who did not adhere to these queries, after three attempts to schedule, were excluded
- By scheduling the patients, it was guaranteed that the waiting time was short
- Responsible participants were provided with reimbursement for travel
- In order to encourage those responsible to continue with their children, the study emphasized the importance of adherence and follow-up, as well as guidelines on oral health and referral for treatment after research

### Statistical analyses

#### Sample size planning

The estimated sample size was calculated based on the primary outcome (child behavior during sedation). Before starting the trial, we considered data from a previous study [9] to estimate the sample size. In the study by Moreira et al. (2013) [9], we found behavior scores (measured using the OSUBRS) associated with the oral administration of midazolam/ketamine (mean 8.6; standard deviation 4.1) and midazolam (mean 14.0; standard deviation 3.8) and no sedative agent (mean 12.5; standard deviation 5.2). Based on these values, 23 cases per group would be required to obtain a power of 80% at the 5% level.

We planned to conduct an interim analysis in April 2016 to monitor the data and to re-estimate the sample size. Hereby, a blinded researcher ran the interim analysis including the first 30 cases. Accordingly, the three groups presented efficacious sedation in 20%, 30%, and 60% of the cases, respectively, considering that, to be an efficacious sedation, a child has to be quiet (OSUBRS score 1) for at least 60% of the session length. Based on the two extremes (20% and 60%), a total of 84 children, or 28 children per group, are needed to fit the criteria for a two-sided significance level of 5% and power of 80%, as calculated by the Fleiss continuity correction method in the OpenEpi version 3. If we had considered the other combinations of values observed in the interim analysis, for example, 20% and 30%, we would need 325 children per group, which would be impracticable for the conclusion of this study, since it would be more difficult to obtain a sample with the strict control of bias (inclusion criteria, procedures, etc.) proposed.

The data needed to analyze the main outcome “child’s behavior” is collected soon after the randomization of the participant, which avoids the problems of loss to follow-up. Similarly, the secondary variables “sedative administration acceptance,” “memory of intraoperative events on the same day,” “salivary cortisol levels,” “intraoperative adverse events,” “pain during treatment,” “caregivers’ and dentists’ perceptions of sedation,” and “cost-analysis” are collected in the same visit. If the caregiver decides to withdraw consent in this same visit and so incomplete data are obtained for the abovementioned variables, the loss will be documented and the participant replaced to reach the final sample of 84.

The collection of data allowing the analysis of the secondary outcomes “memory of intraoperative events 24 h or 1 week later,” “postoperative adverse events” and “child’s perception of dental sedation” need the compliance of the child and caregiver during the follow-up assessments. The participants will be encouraged to continue in the trial through the attentive attitudes of the committed research team (e.g., phone calls and messages), reimbursement of their research expenses (travel, food, etc.) and by emphasizing their awareness of the importance of the investigation. Nevertheless, if they do not complete all secondary measurements, they will not be replaced and their outcomes will be used in an intention-to treat analyses.

#### Data analyses

The intervention will be concealed until after the statistical analysis has been concluded. The data were typed in duplicate and the statistical analysis was performed using statistical software IBM SPSS 24.0 (IBM Corporation, Armonk, NY, USA) and Prism software (GraphPad Prism 6; GraphPad Software, La Jolla, CA, USA), with a significance level of 5%. Descriptive statistics will be provided, overall and by study group. Additionally, bivariate analyses will be performed. Each outcome, primary or secondary, will be considered a dependent variable for distinct statistical bivariate analyses that will be performed separately, as detailed below and in Table 3. The independent variables will be: age, sex, dental history, child caries index, length of visit with sedation and use of protective stabilization during dental sedation, among others.

**Table 3:** Outcome variables and statistical tests

Variable		Outcome measures	Statistical test
Dichotomous	Child’s behavior during the sedation appointment and drug administration	Success of sedation Sedative administration acceptance	Chi-square
	Adverse events occurrence	Intra and postoperative adverse events	Chi-square
	Pain during the sedation (intensity scores will be described)	Sedative administration acceptance Pain during treatment	Chi-square
	Explicit memory	Memory of intraoperative events	Chi-square
Continuous	Salivary cortisol levels	Child’s, parent’s and dentists’ stress	ANOVA or Kruskal-Wallis
	Caregivers’ and dentists’ perception of sedation	Caregivers’ and dentists’ perception of sedation	ANOVA or Kruskal-Wallis

*ANOVA* analysis of variance

The chi-square test will verify the success of sedation based on the child’s behavior (primary outcome) comparing the three groups. Child’s behavior will also be verified according to age subgroups (2–3 years old and 4–6 years old), also comparing the three groups. For the analysis of secondary outcomes, statistical analysis of the variables that need follow-up assessments will be carried out according to the intention-to-treat principle, as explained before. For the continuous variables, the Kolmogorov-Smirnov test will be used to analyze the normality data, and then we will run the analysis of variance (ANOVA) or Kruskal-Wallis tests. A Data Monitoring Committee and external auditing are not required by local standards, because this trial is small, has a short duration and known risks.

## Discussion

The conception of this protocol was based on the following two main goals: to determine a suitable sedative regime for young children undergoing dental treatment and to minimize the biases that have been identified in this type of study, such as lack of a baseline measurement of child’s anxiety and the use of supplemental nitrous oxide [8]. Additionally, we attempted to control for the type of dental procedure to be performed and limit the children’s age range, among other variables. In addition, we will scrutinize the sedation success according to multiple variables and not only the children’s behavior, occurrence of adverse events or completion of the dental treatment. These major points deserve further discussion henceforward.

The goal of the dental sedation for children of 1–6 years old is often solely to complete the treatment [8], but our interest is to accomplish the procedure in the most comfortable way [28]. This explains why we have chosen several outcomes to assess the success of the dental sedation. We had to select one primary outcome that follows the majority of the RCTs on this topic, namely, child behavior. However, we had to propose a new way to assess successful child behavior in an attempt to translate the results of this protocol to a better practice and understanding of the conscious sedation outcomes. Therefore, we chose a valid measure, the OSUBRS, which has been shown to increase the chance of more precise data than scales such as Houpt and Venham for research purposes [29]. It is well known that the lack of a standard behavior assessment scale for trials on pediatric sedation makes it difficult to compare studies [8], but the OSUBRS is one of the most widely used behavior assessment scales for pediatric dental sedation [29], which could favor pooling data in a systematic review. Based on continuous OSUBRS evaluation, we considered that completion of the restorative dental treatment (one tooth) with the child remaining quiet for more than 60% of the duration of the session would represent a satisfactory outcome because they are young, in the preoperational stage [30], and might not be able to cooperate for the whole session even if sedated. There are studies [8] that determine that sedation is successful if it is possible to complete the treatment, regardless of the children’s physical restraint, crying, and struggling.

We also included the occurrence of pain during the drug administration and the dental sedation because the ability to effectively control pain is a crucial aspect of pediatric dentistry. Additionally, we cannot consider a sedative regime successful if it is related to worrisome adverse events, which should be systematically assessed [16].

In addition, if we consider that conscious sedation may not be successful in pediatric dentistry, we need to know if children will remember if they cry/move/struggle during the dental sedation to prevent the induction of trauma. Our research team has developed a systematic review regarding the memory effects of sedative drugs in children [31]; scientific evidence regarding this issue is necessary.

We have already found in a crossover trial (oral midazolam versus placebo, *n* = 18) that a child’s physiological stress response during a sedation appointment, as assessed by salivary cortisol, is not associated with the child’s behavior [27]. Thus, in this RCT, we have planned to verify the cortisol levels in a different study design – parallel, larger sample size, midazolam-ketamine combination, and the oral versus the intranasal route. In a crossover design, it is alleged that the second treatment phase depends on the success of the first treatment period [8].

Another point to be assessed in a potentially successful sedation is the perspective of the people involved – child, parent, and dentist. “Outcome variables need to be more patient-centered” [8]. Therefore, we are investigating the perceptions of the child on the dental sedation session 1 week later as well as the views from the parent who stayed with the child during the procedure and the pediatric dentist who conducted the procedure (quantitative approach). In a qualitative approach, we have already identified that mothers from children sedated for dental treatment self-report stress related to the procedure but are satisfied with this pharmacological method of behavior management [32]. Nevertheless, we have not found a RCT on pediatric dental sedation that examines the children’s view of the procedure.

The other secondary outcome that will allow for a comparison between groups is the sedation costs. It is very important to analyze sedation costs, especially considering public health policies, and these have not received enough attention in pediatric dentistry. In the pediatric ophthalmology field, for example, a cost-effectiveness analysis of clinic-based chloral hydrate sedation versus general anesthesia demonstrated significant savings with sedation but with slightly fewer procedures completed [33]. A systematic review updated in 2015 [34] found no eligible study to compare the morbidity and cost of general anesthesia and sedation for dental treatment in children younger than 18 years.

To participate in this study, children have to present a negative/definitely negative behavior [22] during the dental examination or in a preliminary session in which restorative treatment is performed without sedation. The rationale for this is to avoid including children with positive behavior who were referred for dental treatment under sedation by a dentist who was not able to guide the children’s behavior properly. However, as included children have negative behavior and early childhood caries and many of them require numerous dental procedures, general anesthesia rather than sedation may be the best indication for them. Therefore, one limitation of the present RCT protocol is that we are, in some ways, pushing the limits of sedation indication, although we have restricted the age group involved.

The experimental group will receive a combination of intranasally administered ketamine and midazolam. In fact, intranasal administration of ketamine combined with midazolam has demonstrated a high success rate for child behavior management during dental treatment [11]. However, that study had a crossover design, and the use of an atomizer is not reported, which may interfere with the effect of the sedatives. Additionally, the proposal to first administer ketamine to potentially provide analgesia and minimize the pain associated with intranasally administered midazolam is innovative, as the analgesic effect of intranasally administered ketamine begins as early as 3 min after administration [35]. We chose oral administration of midazolam alone for the control group because it has been shown to improve children’s behavior compared to placebo, according to five heterogeneous studies included in a systematic review, which is the best evidence for a pediatric dental sedation drug, although weak [8].

Blinding is another crucial point for assessing sedation outcomes [8], and we were able to mask the patient/parent, the dental team and the observers to the intervention groups. To achieve that, we have to provide placebos for the sedative route of administration (intranasal and oral) so that all children receive some substance through the nose and mouth. Furthermore, we planned the same sequence of administration for all groups. Additionally, we will videotape the administration of the drugs and the entire dental procedure so that trained and calibrated observers who are not in the dental sedation office can assess the primary outcome. As far as we know, this rigorous masking has not been consistently provided in other RCTs on pediatric dental sedation [8, 36–38]. The video recording of the sedative administration and dental procedures is also original. We propose an action camera adapted for the operator’s head to allow for a close view of the facial expressions of the children, and then, a valid pain assessment through the FLACC [14, 39, 40], which is validated for use with Brazilian children [14].

All in all, this study protocol has strengths that will allow for better analysis of pediatric dental sedation, with a potential impact on health practice and public and private service. Because there is no new medication for sedation in the pipeline, efforts to define a best possible route of administration and combination of drugs is a real need. In addition, the degree of stimulation experienced by patients in the pediatric dental setting makes it a special challenge. This study emphasizes a new and promising protocol in pediatric dentistry combining a feasible route (intranasal) and synergistic drugs.

### Trial status

This RCT began in June 2015 and the process of recruitment is still ongoing.

## Additional files

Additional file 1: World Health Organization Trial Registration Data Set. (PDF 80 kb)

Additional file 2: Consent Forms applied to parents and pediatric dentists (in Portuguese). (PDF 185 kb)

Additional file 3: SPIRIT 2013 Checklist for the NASO study protocol. (DOC 104 kb)

## Abbreviations

ASA: American Society of Anesthesiologists
FLACC: Face, Legs, Activity, Cry, Consolability Pain Assessment Tool
FO: Faculdade de Odontologia
ICER: Incremental cost-effectiveness ratio
NASO: The short form to denominate this investigation (derived from “intranasal”)
NESO: Núcleo de Estudos em Sedação Odontológica
OSUBRS: Ohio State University Behavioral Rating Scale
RCT(s): Randomized controlled trial(s)
SPIRIT: Standard Protocol Items: Recommendations for Interventional Trials
UFG: Universidade Federal de Goiás
VAS: Visual Analogue Scale

## References

[1] Arnrup K, Broberg AG, Berggren U, Bodin L (2007). Temperamental reactivity and negative emotionality in uncooperative children referred to specialized paediatric dentistry compared to children in ordinary dental care. Int J Paediatr Dent.

[2] Klaassen MA, Veerkamp JS, Hoogstraten J (2007). Dental fear, communication, and behavioural management problems in children referred for dental problems. Int J Paediatr Dent.

[3] Taskinen H, Kankaala T, Rajavaara P, Pesonen P, Laitala ML, Anttonen V (2014). Self-reported causes for referral to dental treatment under general anaesthesia (DGA): a cross-sectional survey. Eur Arch Paediatr Dent.

[4] Elledge R, Alexopoulos E, Hosey MT (2007). Short communication: dental anxiety levels and outcomes of care: a preliminary report on experiences of a sedation assessment clinic. Eur Arch Paediatr Dent.

[5] Boyle CA, Newton T, Milgrom P (2009). Who is referred for sedation for dentistry and why?. Br Dent J.

[6] Olley RC, Hosey MT, Renton T, Gallagher J (2011). Why are children still having preventable extractions under general anaesthetic? A service evaluation of the views of parents of a high caries risk group of children. Br Dent J.

[7] American Academy on Pediatric Dentistry Clinical Affairs Committee-Behavior Management Subcommittee. Guideline on behavior guidance for the pediatric dental patient. Pediatr Dent. 2015-2016;37(6):180–93. http://www.aapd.org/media/policies_guidelines/g_behavguide.pdf. Accessed on 25 May 2016. 26531077

[8] Lourenço-Matharu L, Ashley PF, Furness S (2012). Sedation of children undergoing dental treatment. Cochrane Database Syst Rev.

[9] Moreira TA, Costa PS, Costa LR, Jesus-França CM, Antunes DE, Gomes HS (2013). Combined oral midazolam-ketamine better than midazolam alone for sedation of young children: a randomized controlled trial. Int J Paediatr Dent.

[10] Wolfe TR, Braude DA (2010). Intranasal medication delivery for children: a brief review and update. Pediatrics.

[11] Bahetwar SK, Pandey RK, Saksena AK, Chandra G (2011). A comparative evaluation of intranasal midazolam, ketamine and their combination for sedation of young uncooperative pediatric dental patients: a triple blind randomized crossover trial. J Clin Pediatr Dent.

[12] Buonsenso D, Barone G, Valentini P, Pierri F, Riccardi R, Chiaretti A (2014). Utility of intranasal ketamine and midazolam to perform gastric aspirates in children: a double-blind, placebo controlled, randomized study. BMC Pediatr.

[13] Lochary ME, Wilson S, Griffen AL, Coury DL (1993). Temperament as a predictor of behavior for conscious sedation in dentistry. Pediatric Dent.

[14] Silva FC, Thuler LC (2008). Cross-cultural adaptation and translation of two pain assessment tools in children and adolescents. J Pediatr (Rio J).

[15] Ghoneim MM (2004). Drugs and human memory (part 1): clinical, theoretical, and methodologic issues. Anesthesiology.

[16] Mason KP, Green SM, Piacevoli Q, International Sedation Task Force (2012). Adverse event reporting tool to standardize the reporting and tracking of adverse events during procedural sedation: a consensus document from the World SIVA International Sedation Task Force. Br J Anaesth.

[17] Drummond MF, Sculpher MJ, Torrance GW, O’Brien BJ, Stoddart GL (2005). Methods for the economic evaluation of health care programmes.

[18] American Academy of Pediatrics; American Academy of Pediatric Dentistry. Guideline for monitoring and management of pediatric patients during and after sedation for diagnostic and therapeutic procedures. Pediatr Dent. 2015-2016-16;37(6):211–27. Available at: http://www.aapd.org/media/policies_guidelines/g_sedation.pdf. Accessed on 25 May 2016.

[19] Chan AW, Tetzlaff JM, Altman DG, Laupacis A, Gøtzsche PC, Krleža-Jerić K (2013). SPIRIT 2013 Statement: defining standard protocol items for clinical trials. Ann Intern Med.

[20] American Society of Anesthesiologists. ASA Physical Status Classification System. Available at: https://www.asahq.org/resources/clinical-information/asa-physical-status-classification-system. Accessed on 25 May 2016.

[21] Mallampati SR, Gatt SP, Gugino LD, Desai SP, Waraska B, Freiberger D (1985). A clinical sign to predict difficult tracheal intubation: a prospective study. Can Anaesth Soc J.

[22] Frankl S, Shiere F, Fogels H (1962). Should the parent remain with the child in the dental operatory. J Dent Child.

[23] WHO. World Health Organization (2013). Oral health surveys: basic methods.

[24] Ali N, Pruessner JC (2012). The salivary alpha amylase over cortisol ratio as a marker to assess dysregulations of the stress systems. Physiol Behav.

[25] Singh C, Pandey RK, Saksena AK, Chandra G (2014). A comparative evaluation of analgo-sedative effects of oral dexmedetomidine and ketamine: a triple-blind, randomized study. Paediatr Anaesth.

[26] Brasil. Ministério da Saúde. Secretaria-Executiva. Área de Economia da Saúde e Desenvolvimento. Avaliação de tecnologias em saúde: ferramentas para a gestão do SUS / Ministério da Saúde, Secretaria-Executiva, Área de Economia da Saúde e Desenvolvimento. – Brasília: Ministério da Saúde, 2009.

[27] Gomes HS, Corrêa-Faria P, Silva TA, Paiva SM, Costa PS, Batista AC (2015). Oral midazolam reduces cortisol levels during local anaesthesia in children: a randomised controlled trial. Braz Oral Res.

[28] Leroy PL, Costa LR, Emmanouil D, van Beukering A, Franck LS (2016). Beyond the drugs: nonpharmacologic strategies to optimize procedural care in children. Curr Opin Anaesthesiol.

[29] Moura LD, Costa PS, Costa LR (2016). How do observational scales correlate the ratings of children’s behavior during pediatric procedural sedation?. Biomed Res Int.

[30] Feigal RJ (2001). Guiding and managing the child dental patient: a fresh look at old pedagogy. J Dent Educ.

[31] Viana KA, Daher A, Maia LC, Costa PS, Martins CC, Paiva SM (2016). Memory effects of sedative drugs in children and adolescents-protocol for a systematic review. Syst Rev.

[32] Lima ARA, Medeiros M, Costa LR (2015). Mothers’ perception about pediatric dental sedation as an alternative to dental general anesthesia. RGO. Rev Gauch Odontol.

[33] Burnett HF, Lambley R, West SK, Ungar WJ, Mireskandari K (2015). Cost-effectiveness analysis of clinic-based choral hydrate sedation versus general anaesthesia for paediatric ophtalmological procedures. Br J Ophthalmol.

[34] Ashley PF, Williams CE, Moles DR, Parry J (2015). Sedation versus general anaesthesia for provision of dental treatment to patients younger than 18 years. Cochrane Database Syst Rev.

[35] Johansson J, Sjöberg J, Nordgren M, Sandström E, Sjöberg F, Zetterström H (2013). Prehospital analgesia using nasal administration of S-ketamine—a case series. Scand J Trauma Resusc Emerg Med.

[36] Shanmugaavel AK, Asokan S, Baby JJ, Priya G, Gnana DJ (2016). Comparison of behavior and dental anxiety during intranasal and sublingual midazolam sedation—a randomized controlled trial. J Clin Pediatr Dent.

[37] Sunbul N, Delvi MB, Zahrani TA, Salama F (2014). Buccal versus intranasal midazolam sedation for pediatric dental patients. Pediatr Dent.

[38] Tyagi P, Tyagi S, Jain A (2013). Sedative effects of oral midazolam, intravenous midazolam and oral diazepam in the dental treatment of children. J Clin Pediatr Dent.

[39] Merkel SI, Voepel-Lewis T, Shayevitz JR, Malviya S (1997). The FLACC: a behavioral scale for scoring postoperative pain in young children. Pediatr Nurs.

[40] Crellin DJ, Harrison D, Santamaria N, Babl FE (2015). Systematic review of the Face, Legs, Activity, Cry and Consolability Scale for assessing pain in infants and children: is it reliable, valid, and feasible for use?. Pain.

[41] International Committee of Medical Journal Editors. Defining the role of authors and contributors. Available in: http://www.icmje.org/recommendations/browse/roles-and-responsibilities/defining-the-role-of-authors-and-contributors.html. Accessed on 18 March 2017.

