# Supplementary material for: Intranasal sedation using ketamine and midazolam for pediatric dental treatment (NASO): study protocol for a randomized controlled trial
Source: Trials. 2017 Apr 11;18:172. doi: 10.1186/s13063-017-1919-2 (PMC5387269; doi:10.1186/s13063-017-1919-2)
Supplement: Supplementary file 1 — World Health Organization Trial Registration Data Set. (PDF 80 kb) [file 13063_2017_1919_MOESM1_ESM.pdf]

ClinicalTrials.gov Protocol Registration and Results System (PRS) Receipt
Release Date: March 20, 2017

ClinicalTrials.gov ID: NCT02447289

Study Identification

Unique Protocol ID: 041530

Brief Title: Midazolam and Ketamine Effect Administered Through the Nose for Sedation of Children for Dental Treatment (NASO)

Official Title: Efficacy, Safety and Cost-effectiveness of Intranasal Sedation With Ketamine and Midazolam in Pediatric Dentistry: a Randomized Clinical Trial

Secondary IDs:

Study Status

Record Verification: March 2017

Overall Status: Active, not recruiting

Study Start: June 2015 []

Primary Completion: December 2016 [Actual]

Study Completion: July 2017 [Anticipated]

Sponsor/Collaborators

Sponsor: Universidade Federal de Goias

Responsible Party: Principal Investigator
Investigator: Luciane Ribeiro de Rezende Sucasas da Costa [lcosta]
Official Title: Professor
Affiliation: Universidade Federal de Goias

Collaborators: Conselho Nacional de Desenvolvimento Científico e Tecnológico

Oversight

U.S. FDA-regulated Drug:

U.S. FDA-regulated Device:

IND/IDE Protocol: No

Human Subjects Review: Board Status: Approved
Approval Number: 36411214.1.0000.5083
Board Name: UFG Research Ethics Committee
Board Affiliation: National Committee of Ethics in Research
Phone: +55(62)3521-1215
Email: cep.prpi.ufg@gmail.com
Address:

Pró-Reitoria de Pesquisa e Inovação, Universidade Federal de Goiás, Caixa Postal: 131, Prédio da Reitoria, Piso 1, Campus II (Samambaia). CEP: 74001-970, Goiânia, Goiás, Brasil.

Data Monitoring: No

Plan to Share IPD: Undecided
IPD can be available as requested by the scientific journal where the manuscript is gonna be submitted

FDA Regulated Intervention: No

Study Description

Brief Summary: Randomised clinical trial to test the efficacy, safety and cost-effectiveness of sedatives; masked, controlled, parallel design with three arms, phase 4.

Detailed Description: About 10% of children may have behavioral problems during dental treatment, but the evidence for sedative protocols that benefit the care in pediatric dentistry are still weak. This randomized, masked, controlled, parallel design study aims to evaluate the efficacy, safety and cost-effectiveness of intranasal sedation with ketamine / midazolam in healthy children aged 2 to 6 years old. Children will be selected among those with dental decay that do not allow dental treatment to be performed, and need at least two restorations under local anesthesia and rubber dam isolation. It is estimated a preliminarily a sample of 84 children equally divided into three groups: A) Ketamine (4.0 mg / kg up to 100 mg) + midazolam (0.2 mg / kg, maximum 5 mg) intranasally; B) ketamine (4.0 mg / kg up to 100 mg) + midazolam (0.5 mg / kg, maximum 20 mg) orally; C) midazolam (1.0 mg / kg, maximum 20 mg) orally. The hypothesis is that ketamine / midazolam combination, administered intranasally, it constitutes an effective sedative regime, safe and cost-effective for use in pediatric dentistry in the public service. The primary endpoint is the behavior of the child. Secondary endpoints are: sedative administration acceptance, procedure memory and salivary cortisol levels within each intervention group. Additionally, we will evaluate: the occurrence of pain and associated changes in facial expressions with potentially painful stimuli in children sedated for dental care; caregivers and paediatric dentists' stress and perception about sedation; adverse events that occur during and after administration of sedatives; cost-effectiveness of the sedative protocols. The experimental design is planned to minimize systematic and random errors and to contribute to higher level of evidence in future systematic reviews. The outcomes of this study have potential impact on public and private health practice, and may support institutional guidelines dealing with this theme.

Conditions

Conditions: Child Behavior
Dental Caries

Keywords: Dental caries
Moderate sedation

Study Design

Study Type: Interventional

Primary Purpose: Treatment

Study Phase: Phase 4

Interventional Study Model: Parallel Assignment

Number of Arms: 3

Masking: Participant, Care Provider, Investigator, Outcomes Assessor

Allocation: Randomized

Enrollment: 84 [Actual]

Arms and Interventions

Arms	Assigned Interventions
Experimental: Experimental<br>Intranasal administration of ketamine (4.0 mg/kg, max. 100 mg) and midazolam (0.2 mg/kg, max. 5 mg)	Drug: Intranasal ketamine<br>Drug: Intranasal midazolam
Active Comparator: Comparator<br>Oral administration of ketamine (4.0 mg/kg, max. 100 mg) and midazolam (0.5 mg/kg, max. 20 mg)	Drug: Oral ketamine<br>Drug: Oral midazolam
Active Comparator: Control<br>Oral administration of midazolam (1.0 mg/kg, max. 20 mg)	Drug: Oral midazolam

Outcome Measures

Primary Outcome Measure:

1. Child's behavior measured by an observational scale
   Children's behavior assessed by OSUBRS (Ohio State University Behavioral Rating Scale) in digital videos recorded during the administration. Scores: 1 - quiet, 2 - cry with movement, 3 - movement without cry; 4 - struggling. Satisfactory behavior means more than 60% of score 1 during the entire appointment.

   [Time Frame: Participants will be followed for the duration of the dental session, an expected average of 40 minutes]

Secondary Outcome Measure:

2. Acceptance of sedative administration measured by an observational scale
   Acceptance of the sedative administration, assessed by OSUBRS (Ohio State University Behavioral Rating Scale) minute by minute in digital videos recorded during the administration. Scores: 1 - quiet, 2 - cry with movement, 3 - movement without cry; 4 - struggling.

   [Time Frame: Participants will be assessed for the duration of the sedative administration, an expected average of 3 minutes]

3. Participants' stress according to salivary cortisol
   Occurrence of stress during dental procedure, assessed by salivary cortisol in children, their accompanying parent and the dentist in charge; the ELISA test will determine changes in cortisol level during dental treatment

   [Time Frame: Change from baseline in the salivary cortisol at an expected average of 40 minutes]

4. Pain measured by the FLACC Pain Assessment Tool
   Occurrence of pain and associated changes in body expressions with potentially painful stimuli in children sedated for dental care

   [Time Frame: Participants will be followed in video files for the duration of the dental session, an expected average of 40 minutes]

5. Caregivers' and dentists' perceptions on sedation measured by a Visual Analogue Scale
   Perception of caregivers and dentists on sedation, through self-report

   [Time Frame: During and soon after the dental session]

6. Child's perceptions on sedation through a semi-structured interview
   Child's perception concerning the dental treatment under sedation

[Time Frame: One week after the dental session]

7. Memory of the dental procedure at the immediate post-operative period measured by a validated test
   Memory of the dental procedure, according to test with figures validated in Brazil. Amnesia will be reported if children do not recall the figures showed after sedative administration

   [Time Frame: Participants will be assessed for the recall of the dental procedure, at a time point of 20 minutes (average) after the end of the dental procedure]

8. Memory of the dental procedure after 24 hours measured by a validated test
   Memory of the dental procedure, according to test with figures validated in Brazil. Amnesia will be reported if children do not recall the figures showed after sedative administration

   [Time Frame: Participants will be assessed for the recall of the dental procedure, at a time point of 24 hours (average) after the end of the dental procedure]

9. Adverse events during the dental procedure
   Occurrence of adverse events assessed by the World SIVA tool; adverse events will be categorised into minor, sentinel and major

   [Time Frame: Participants will be followed for the duration of the dental session, an expected average of 40 minutes]

10. Post-operative adverse events
    Occurrence of adverse events while child is in the recovery room and after discharge, assessed by the World SIVA tool; adverse events will be categorised into minor, sentinel and major

    [Time Frame: 24 hours]

11. Cost analysis
    Cost-effectiveness of different sedation protocols according to the methodology of health technologies assessment; the cost of each intervention will be compared

    [Time Frame: One year and a half]

Eligibility

Minimum Age: 2 Years

Maximum Age: 6 Years

Sex: All

Gender Based:

Accepts Healthy Volunteers: Yes

Criteria: Inclusion Criteria:

- American Society of Anesthesiologists I (healthy) or II (mild and controlled systemic disease such as persistent asthma)
- Small risk of airway obstruction (Mallampati less than 2 or tonsillar hypertrophy occupying less than 50% of the oropharynx)
- No medical history of neurological or cognitive changes
- Absence of facial deformities
- Term birth
- No use of drugs that may impair cognitive functions
- At least two teeth with cavities without pulp involvement, requiring dental restoration under local anesthesia and rubber dam

Exclusion Criteria:

- Positive or definitely positive behavior (Frankl et al, 1962) in a dental treatment session conducted by the research team

Contacts/Locations

Central Contact Person: Paulo S Costa, DDS, MS, PhD
Telephone: +556232096151
Email: paulosucasas@ufg.br

Central Contact Backup:

Study Officials: Luciane R Costa, DDS, MS, PhD
Study Principal Investigator
Professor

Locations: Brazil
Dental School
Goiania, Goias, Brazil, 74605220
Contact: Luciane R Costa, DDS, MS, PhD 55 62 32096047
lsucasas@ufg.br
Sub-Investigator: Paulo S Sucasas, MD, MS, PhD

References

Citations:

Links:

Study Data/Documents:

U.S. National Library of Medicine | U.S. National Institutes of Health | U.S. Department of Health & Human Services
