# Supplementary material for: Intranasal sedation using ketamine and midazolam for pediatric dental treatment (NASO): study protocol for a randomized controlled trial
Source: Trials. 2017 Apr 11;18:172. doi: 10.1186/s13063-017-1919-2 (PMC5387269; doi:10.1186/s13063-017-1919-2)
Supplement: Supplementary file 2 — Consent Forms applied to parents and pediatric dentists (in Portuguese). (PDF 185 kb) [file 13063_2017_1919_MOESM2_ESM.pdf]

CONSENT FORM – PARENT ON BEHALF OF CHILD

FACULDADE DE ODONTOLOGIA UFG

PROJETO NASO

Termo de Consentimento Livre e Esclarecido – CRIANÇA

Participante
N___

Você é convidada(o) a autorizar que seu filho(a) ____ participe da pesquisa "**Eficácia, segurança e custo-efetividade da sedação intranasal com cetamina e midazolam em odontopediatria: ensaio clínico randomizado**", realizada no Núcleo de Estudos em Sedação Odontológica (NESO) da Faculdade de Odontologia da UFG. Meu nome é ____; faço parte da equipe que desenvolve esta pesquisa. Após ler este termo com atenção, ser esclarecida(o) sobre suas informações, ter retirado todas as suas dúvidas, e caso aceite fazer parte do estudo, por favor, assine este documento, que está em duas vias, sendo que uma será para você e a outra para os pesquisadores. Em caso de dúvidas sobre a pesquisa, você pode me procurar pelo telefone _________________ ou a Profa. Luciane Costa pelo telefone XXXXXXX (inclusive com ligações a cobrar). Em caso de dúvidas sobre os seus direitos como participante nessa pesquisa, você poderá entrar em contato com o Comitê de Ética em Pesquisa da UFG pelo telefone (62) 3521-1215.

INFORMAÇÕES SOBRE A PESQUISA:

Pesquisadora Responsável: Profa. Dra. Luciane R. R. S. Costa (odontopediatra)

Demais pesquisadores: Paulo Sérgio Sucasas da Costa (médico pediatra), Aline Carvalho Batista (patologista bucal), Anelise Daher Vaz Castro (odontopediatra), Mara Rúbia de Camargo Alves Orsini (psicóloga), Liliani Aires Cândido Vieira (odontopediatra), Geovanna de Castro Morais Machado (odontopediatra), Heloísa Sousa Gomes (cirurgiã-dentista), Daniella Miranda (psicóloga), Larissa da Silva Moura (odontopediatra), Karolline Alves Viana (cirurgiã-dentista), Joji Sado Filho (médico residente em Anestesiologia) , Mônica Maia Moterane (aluna do curso de graduação em Odontologia), Nayara Rúbia de Araújo (aluna do curso de graduação em Medicina), Jordana Guedes de Amorim Mendonça (aluna do curso de graduação em Odontologia).

Estamos fazendo esta pesquisa porque, algumas vezes, crianças muito jovens não deixam o dentista tratar os dentes com cárie porque são ansiosas e/ou imaturas. Nesta pesquisa, queremos saber quais remédios podem relaxar mais a criança durante o tratamento.

Caso você aceite participar da pesquisa, uma dentista especialista em crianças irá limpar e examinar os dentes do seu filho e um médico vai ver como está a saúde dele. Em outro dia, um dente com cárie será tratado com anestesia local, sem usar remédio para acalmar. A dentista vai acalmar a criança de outras maneiras, conversando, cantando e distraindo seu filho. Se ele deixar tratar os dentes, o resto do tratamento será feito sem precisar tomar remédio. Se ele não deixar tratar os dentes, será marcado para outro dia para

tomar remédio e tratar outro dente com cárie. Os remédios serão dados pela boca e pelo nariz, sendo que o médico é quem vai decidir sobre isso. Todos os remédios já foram testados antes e são seguros quando usados corretamente. Durante o tratamento, seu filho será filmado para que possamos depois, com calma, avaliar como ele se comportou. As imagens dele só serão usadas para apresentação em aulas ou congressos se você concordar.

Durante o tratamento, será colhida a saliva de seu filho para ver se ele está estressado. Algumas figuras e brinquedos vão ser mostrados para seu filho antes do tratamento, para ver se depois ele se lembra quais eram. Vamos te telefonar no dia seguinte para saber como seu filho está passando. A conversa por telefone será gravada. Uma semana depois, você deverá retornar à clínica para que a criança seja entrevistada para contar o que ele achou do tratamento.

O batimento do coração e a respiração do seu filho serão checados a todo momento. Problemas depois de tomar o remédio para acalmar quase não acontecem, mas pode ser que seu filho apresente as seguintes reações: enjoo, vômito, agitação, alucinação, dificuldade de respirar, tosse, alergia ao remédio, sono exagerado, queda de pressão, alteração na batida do coração, parada cardíaca, sono profundo. Se algum desses problemas acontecer, os médicos irão socorrer seu filho imediatamente. Pode ser que algumas horas depois do atendimento, em casa, seu filho também fique agitado, tenha enjoo, vômitos, tonturas, mal-estar, dificuldade de respirar ou muito sono. Se isso acontecer, você poderá ligar para os telefones da equipe, para se informar sobre o que fazer para seu filho melhorar.

Antes da consulta em que seu filho receberá o remédio, você responderá alguns questionários sobre dor, ansiedade e qualidade de vida. Depois que seu filho terminar todo tratamento dentário, telefonaremos para você (duas semanas após e três meses após) para saber como está a qualidade de vida de vocês.

Você poderá receber dinheiro para despesas com transporte ou alimentação, caso precise. Se você não quiser participar, nada acontecerá a você e isso não impedirá que seu filho faça o tratamento. Se você achar que alguma coisa ruim aconteceu à criança por causa da pesquisa, poderá pedir uma indenização. Sempre que precisar, poderá tirar suas dúvidas com a pesquisadora. Se você quiser desistir de participar após dar seu aceite, também não será prejudicado.

Os resultados desta pesquisa poderão ajudar, no futuro, outras crianças que também tenham medo de tratar de dentes e que vão precisar tomar remédio para ficarem calmas.

____

Luciane Ribeiro de Rezende Sucasas da Costa

Pesquisadora responsável

CONSENTIMENTO DO USO DE IMAGENS DA CRIANÇA

Eu autorizo a equipe da pesquisa a usar as imagens do meu filho, obtidas nas filmagens durante o tratamento odontológico, para mostrar a pesquisa em aulas ou em congressos científicos.

( ) Sim. Assinatura:_____________________ ( ) Não. Assinatura:_____________________

CONSENTIMENTO DA PARTICIPAÇÃO DA CRIANÇA

Eu, ____,
RG/CPF_____________________________, responsável por ______________________________________,
autorizo sua participação na pesquisa **Eficácia, segurança e custo-efetividade da sedação intranasal com cetamina e midazolam em odontopediatria: ensaio clínico randomizado**. Fui devidamente informado(a) e esclarecido(a) pelos pesquisadores sobre a pesquisa, sobre o que vai ser feito, os possíveis benefícios e riscos e sobre o ressarcimento decorrente da participação da criança. Foi-me garantido que posso desistir a qualquer momento sem que o tratamento do meu filho seja interrompido.

Assinatura do Responsável: _____________________________________Goiânia, __/__/___

Impressão datilosópica

Presenciamos a solicitação de consentimento, esclarecimentos sobre a pesquisa e o aceite do participante.

Testemunhas:

1) Nome: ____
Assinatura:____
R.G.:__________________________

2) Nome: ____
Assinatura:____
R.G.:__________________________

CONSENT FORM – ACCOMPANYING PERSON

FACULDADE DE ODONTOLOGIA UFG

PROJETO NASO

Termo de Consentimento Livre e Esclarecido - ACOMPANHANTE

**Participante**
**N___**

Você é convidada(o) a participar da pesquisa "**Eficácia, segurança e custo-efetividade da sedação intranasal com cetamina e midazolam em odontopediatria: ensaio clínico randomizado**", realizada no Núcleo de Estudo em Sedação Odontológica (NESO) da Faculdade de Odontologia da UFG. Meu nome é ____; faço parte da equipe que desenvolve esta pesquisa. Após ler este termo com atenção, ser esclarecida(o) sobre suas informações, ter retirado todas as suas dúvidas, e caso aceite fazer parte do estudo, por favor, assine este documento, que está em duas vias, sendo que uma será para você e a outra para os pesquisadores. Em caso de dúvidas sobre a pesquisa, você pode me procurar pelo telefone _________________ ou a Profa. Luciane Costa pelo telefone XXXXXXX (inclusive com ligações a cobrar). Em caso de dúvidas sobre os seus direitos como participante nessa pesquisa, você poderá entrar em contato com o Comitê de Ética em Pesquisa da UFG pelo telefone (62) 3521-1215.

INFORMAÇÕES SOBRE A PESQUISA:

Pesquisadora Responsável: Profa. Dra. Luciane R. R. S. Costa (odontopediatra)

Demais pesquisadores: Paulo Sérgio Sucasas da Costa (médico pediatra), Aline Carvalho Batista (patologista bucal), Anelise Daher Vaz Castro (odontopediatra), Mara Rúbia de Camargo Alves Orsini (psicóloga), Liliani Aires Cândido Vieira (odontopediatra), Geovanna de Castro Morais Machado (odontopediatra), Heloísa Sousa Gomes (cirurgiã-dentista), Daniella Miranda (psicóloga), Larissa da Silva Moura (odontopediatra), Karolline Alves Viana (cirurgiã-dentista), Joji Sado Filho (médico residente em Anestesiologia) , Mônica Maia Moterane (aluna do curso de graduação em Odontologia), Nayara Rúbia de Araújo (aluna do curso de graduação em Medicina), Jordana Guedes de Amorim Mendonça (aluna do curso de graduação em Odontologia).

Estamos fazendo esta pesquisa porque, algumas vezes, crianças muito jovens não deixam o dentista tratar os dentes com cárie porque são ansiosas e/ou imaturas. Nesta pesquisa, queremos saber quais remédios podem relaxar mais a criança durante o tratamento.

Caso você aceite fazer parte da pesquisa, sua saliva será colhida com rolinho de algodão durante o atendimento do seu filho, para vermos se você está estressado. Faremos ainda, duas perguntas a você sobre o que achou do seu filho ter tomado o remédio para ficar calmo(a). A sua saliva será usada apenas nesta pesquisa, e tomaremos cuidado para que seu nome não seja exposto para outras pessoas que não façam parte da pesquisa.

Você poderá receber dinheiro para despesas com transporte ou alimentação, caso precise. Se você não quiser participar, nada acontecerá a você e isso não impedirá que seu filho faça o tratamento. Se você achar que alguma coisa ruim aconteceu a você por causa da pesquisa, poderá pedir uma indenização. Sempre que precisar, poderá tirar suas dúvidas com a pesquisadora. Se você quiser desistir de participar após dar seu aceite, também não será prejudicado.

Os resultados desta pesquisa poderão ajudar, no futuro, outras crianças que também tenham medo de tratar de dentes e que vão precisar tomar remédio para ficarem calmas.

____

Luciane Ribeiro de Rezende Sucasas da Costa

Pesquisadora responsável

CONSENTIMENTO DA PARTICIPAÇÃO DO RESPONSÁVEL

Eu, ____, RG/CPF_____________________________, aceito ser participante da pesquisa **Eficácia, segurança e custo-efetividade da sedação intranasal com cetamina e midazolam em odontopediatria: ensaio clínico randomizado**. Fui devidamente informado(a) e esclarecido(a) pelos pesquisadores sobre a pesquisa, sobre o que vai ser feito, os possíveis benefícios e riscos e sobre o ressarcimento decorrente da minha participação. Foi-me garantido que posso desistir a qualquer momento sem que o tratamento do meu(minha) filho(a) seja interrompido.

Assinatura do Responsável: ____________________________________Goiânia, __/__/___

Impressão datilosópica

Presenciamos a solicitação de consentimento, esclarecimentos sobre a pesquisa e o aceite do participante.

Testemunhas:

1) Nome: ____
   Assinatura:____
   R.G.:__________________________
2) Nome: ____
   Assinatura:____
   R.G.:__________________________

CONSENT FORM – PEDIATRIC DENTIST

FACULDADE DE ODONTOLOGIA UFG

PROJETO NASO

Termo de Consentimento Livre e Esclarecido – ODONTOPEDIATRA

Participante
N___

Você é convidada(o) a participar como odontopediatra da pesquisa "**Eficácia, segurança e custo-efetividade da sedação intranasal com cetamina e midazolam em odontopediatria: ensaio clínico randomizado**", realizada no Núcleo de Estudo em Sedação Odontológica (NESO) da Faculdade de Odontologia da UFG. Meu nome é ____, e faço parte da equipe desta pesquisa. Após ler este termo com atenção, ser esclarecida(o) sobre suas informações, ter retirado todas as suas dúvidas, e caso aceite fazer parte do estudo, assine este documento, que está em duas vias, sendo que uma será para você e a outra para os pesquisadores. Em caso de dúvidas sobre a pesquisa, você pode me procurar pelo telefone __________________ ou a Profa. Luciane Costa pelo telefone XXXXXX (inclusive com ligações a cobrar). Em caso de dúvidas sobre os seus direitos como participante nessa pesquisa, você poderá entrar em contato com o Comitê de Ética em Pesquisa da UFG pelo telefone (62) 3521-1215.

INFORMAÇÕES SOBRE A PESQUISA:

Pesquisadora Responsável: Profa. Dra. Luciane R. R. S. Costa (odontopediatra)

Demais pesquisadores: Paulo Sérgio Sucasas da Costa (médico pediatra), Aline Carvalho Batista (cirurgiã-dentista), Anelise Daher Vaz Castro (odontopediatra), Mara Rúbia de Camargo Alves Orsini (psicóloga), Liliani Aires Cândido Vieira (odontopediatra), Geovanna de Castro Morais Machado (odontopediatra), Heloísa Sousa Gomes (cirurgiã-dentista), Daniella Miranda (psicóloga), Larissa da Silva Moura (odontopediatra), Karolline Alves Viana (cirurgiã-dentista), Joji Sado Filho (médico residente em Anestesiologia) , Mônica Maia Moterane (Aluna do curso de graduação em Odontologia), Nayara Rúbia de Araújo (Aluna do curso de graduação em Medicina) Jordana Guedes de Amorim Mendonça (Aluna do curso de graduação em Odontologia).

Crianças de até 6 anos de idade podem resistir ao tratamento odontológico devido ao medo/ansiedade e por não ter o desenvolvimento necessário para compreender a necessidade do tratamento. O objetivo deste trabalho é investigar regimes sedativos que beneficiem o tratamento odontológico de crianças pré-escolares.

Caso você aceite fazer parte da pesquisa, sua saliva será coletada em vários momentos do atendimento, para verificação do seu nível de estresse. Você ainda será indagado, através de duas perguntas, sobre sua percepção a respeito da sedação da criança. A saliva coletada será passível de ser identificada, porém os pesquisadores apenas a utilizarão para o objetivo desta pesquisa. Os resultados deste estudo têm o potencial de beneficiar crianças que se assemelhem às condições desta amostra na medida em que um regime sedativo eficiente for identificado.

Ao término de cada sessão de atendimento e sempre que achar necessário, você poderá tirar suas dúvidas com a pesquisadora. Você receberá ressarcimento para participar da pesquisa, caso necessite. Você tem a nossa garantia de que o seu não consentimento não lhe trará nenhum prejuízo. Você poderá solicitar indenização em caso de danos que sejam comprovadamente decorrentes de sua participação nessa pesquisa.

____

Luciane Ribeiro de Rezende Sucasas da Costa

Pesquisadora responsável

CONSENTIMENTO DA PARTICIPAÇÃO DA ODONTOPEDIATRA

Eu, ____, RG/CPF__________________________________, aceito ser participante do estudo **Eficácia, segurança e custo-efetividade da sedação intranasal com cetamina e midazolam em odontopediatria: ensaio clínico randomizado**. Fui devidamente informado(a) e esclarecido(a) pelos pesquisadores sobre a pesquisa, sobre os procedimentos nela envolvidos, assim como os possíveis benefícios e riscos e ressarcimento decorrentes da minha participação. Foi-me garantido que posso retirar meu consentimento a qualquer momento e não levará a qualquer penalidade.

Assinatura do(a) Odontopediatra: ____

Goiânia, ____/____/_______
